# Effect of a digital tool on breast cancer specific health-related quality of life and psychological distress: secondary results from the ADAPT study

**DOI:** 10.1007/s00520-025-09968-8

**Published:** 2025-10-08

**Authors:** Noelle J. M. C. Vrancken Peeters, Sofia Georgopoulou, Rafal Kulakowski, Emma Hainsworth, Emma Lidington, Sophie E. McGrath, Jillian Noble, Leyla Azarang, Olga Husson, Susanne Cruickshank

**Affiliations:** 1 Department of Medical Oncology, Netherlands Cancer Institute – Antoni van Leeuwenhoek Hospital, 1066 CX Amsterdam, The Netherlands; 2 Erasmus MC Cancer Institute, Erasmus Medical Centre, Rotterdam, The Netherlands; 3 The Royal Marsden Hospital, London, UK; 4 Imperial College Health Partners, London, UK; 5 University of Stirling, Stirling, UK

**Keywords:** Breast cancer, EHealth, Health-related quality of life (HRQoL), Psychological distress, Supportive care

## Abstract

**Purpose:**

This report presents the secondary outcomes of the ADAPT trial, focusing on the impact of a digital tool designed to support people affected by breast cancer, on breast cancer-specific health-related quality of life (HRQoL) and psychological distress over the first year from diagnosis.

**Methods:**

Women with early-stage breast cancer were randomly assigned (1:1) to standard care (control) or standard care plus the breast cancer digital tool (intervention). Data were collected using a demographic questionnaire, the European Organization for Research and Treatment of Cancer Core Quality of Life questionnaire BR45 (EORTC QLQ-BR45) for breast cancer-specific HRQoL, and the Hospital Anxiety and Depression Scale (HADS) for psychological distress at baseline, 6 weeks, 3 months, 6 months, and 1 year from diagnosis. Linear mixed effect model regression was used to assess the effect of the digital tool over time while correcting for intra-participant correlation.

**Results:**

There were no significant differences (*p* > 0.05) in the EORTC QLQ-BR45 scales and items and HADS scores between participants in the intervention and control groups when adjusted for the primary tumor stage.

**Conclusion:**

The breast cancer digital tool did not have a significant effect on breast cancer–specific HRQoL and psychological distress over time. The results are consistent with the findings of the primary ADAPT paper, which demonstrated that the digital tool did not have a significant effect on patient activation, HRQoL, and health status*.* There is a need for personalized support and strategies to enhance digital tool adherence, offering more customized self-management approaches for people affected by breast cancer in the future.

**Trial registration:**

The study was registered at https://clinicaltrials.gov (NCT03866655) on 7 March 2019 (https://clinicaltrials.gov/study/NCT03866655). The full protocol is accessible through the following link: https://trialsjournal.biomedcentral.com/articles/10.1186/s13063-019-3971-6.

## Introduction

Breast cancer, the most prevalent cancer among women worldwide, has evolved into a chronic disease due to advancements in new therapies and early detection, which consequently has increased the need for more effective long-term care and support [1–6]. However, existing breast cancer treatments and interventions do not address all the long-term care needs of individuals affected by breast cancer [7]. As a result, encouraging self-care among those affected by breast cancer has become increasingly important.

The ADAPT randomized controlled trial (RCT) examined a breast cancer digital tool focused on self-management and designed to help people affected by breast cancer regain control after a diagnosis. The app provides needs-tailored medical information, self-monitoring of symptoms via a tracker, and functions to self-manage care, such as an appointment calendar, a modifiable question list, and a consultation recording device [8, 9].

The primary paper of the ADAPT study demonstrated that although positive trends were noted, the digital app did not have a significant effect on patient activation, health-related quality of life (HRQoL), and health status over time compared to standard care alone in participants with early-stage breast cancer [9]. This report aims to present the secondary outcomes of the ADAPT trial concerning the impact of the digital tool on breast cancer-specific HRQoL and psychological distress over the first year from diagnosis [8].

## Methods

CONSORT-EHEALTH (Consolidated Standards of Reporting Trials of Electronic and Mobile HEalth Applications and onLine TeleHealth) was followed when reporting the results of this RCT [10]. The detailed methodology of this study is described in the study protocol, covering the breast cancer digital tool intervention, its preliminary pilot testing, participant criteria, study design, randomization, data collection methods, outcome measures, sample size estimation, and statistical analysis plan [8]. The baseline characteristics of the study population (*n* = 166), as well as the primary outcome, have been previously detailed in the initial ADAPT paper [9].

### Study design and methods

#### Study design

The ADAPT study was a multi-center, individually randomized, parallel-controlled trial (*n* = 166). Women (≥ 18 years) with primary early-stage breast cancer receiving care at one of the participating sites were randomly assigned (1:1) to one of the study groups: the intervention or the control group. The control group received standard care, and the intervention group received standard care in addition to the digital app [8].

#### Data collection

Participants completed a questionnaire package at baseline (T0) before starting anti-cancer treatment, which included participant-reported sociodemographic information and validated questionnaires such as the European Organization for Research and Treatment of Cancer Core Quality of Life questionnaire BR45 (EORTC QLQ-BR45) and the Hospital Anxiety and Depression Scale (HADS) [11, 12]. A follow-up questionnaire package was sent to the participants 6 six weeks (T1), 3 months (T2), 6 months (T3), and 1 year (T4) after diagnosis. This package included the same information as the baseline questionnaire package, with a shorter version of the socio-demographic section.

Clinical information was extracted from the electronic patient records (EPR) of the hospital where the participants were treated. This information was captured using an electronic case report form (CRF) and subsequently stored in a central database [8].

### Outcomes

#### Breast cancer-specific HRQoL assessment

Breast cancer–specific HRQoL was assessed using the EORTC QLQ-BR45, which includes five functional scales or items (body image, future perspective, sexual functioning, sexual enjoyment, and breast satisfaction) and seven symptom scales or items (systemic therapy side effects, upset by hair loss, arm symptoms, breast symptoms, endocrine therapy symptoms, skin mucosis symptoms, and endocrine sexual symptoms). Scales are calculated based on the guidelines from the EORTC. After linear transformation, all scales and single-item measures range in score from 0 to 100. A higher score on the functional scales and items indicates better functioning and HRQoL. A higher score on the symptom scales and items indicates a higher symptom burden [11, 13, 14].

#### Psychological distress assessment

Psychological distress was assessed using the HADS. This 14-item instrument measures psychological distress on a 4-point Likert scale (range 0–3), with seven items assessing anxiety and seven items assessing depression. For anxiety and depression, the total score is the sum of the respective seven items (ranging from 0 to 21). The summed total score of anxiety and depression reflects the overall level of psychological distress (ranging from 0 to 42). In total, three continuous scales can be calculated (anxiety, depression, and overall psychological distress). Higher total scores are indicative of more psychological.

### Statistical analysis

Linear mixed effect models were used to analyze the effect of the digital tool on HRQoL and psychological distress over time. To examine the trend of the scales, items, and scores at each consecutive time interval, time was considered as a categorical covariate in the model (timepoint). The effect of time was specified as a fixed effect, and participant ID as the grouping factor. An interaction term between time and study group was included in the models. The baseline effect of the study groups was not included due to the randomization process. Baseline characteristics that were significantly different between participants in the intervention and control group were considered as fixed effects (primary tumor stage). Effect plots were created to visualize the impact of the digital tool on HRQoL and psychological distress over time for a participant with the most common baseline characteristics [8, 9].

A two-sided *p*-value of 0.05 was considered statistically significant. All statistical analyses were performed using R version 4.4.2 (R Development Core Team and the R Foundation for Statistical Computing) by integrating software from open-source packages, including “nlme” and packages from the “tidyverse” such as “dplyr”, “tidyr”, and “ggplot2” [8, 9, 15–17].

## Results

### EORTC QLQ-BR45: functional scales and items

**Fig. 1:**
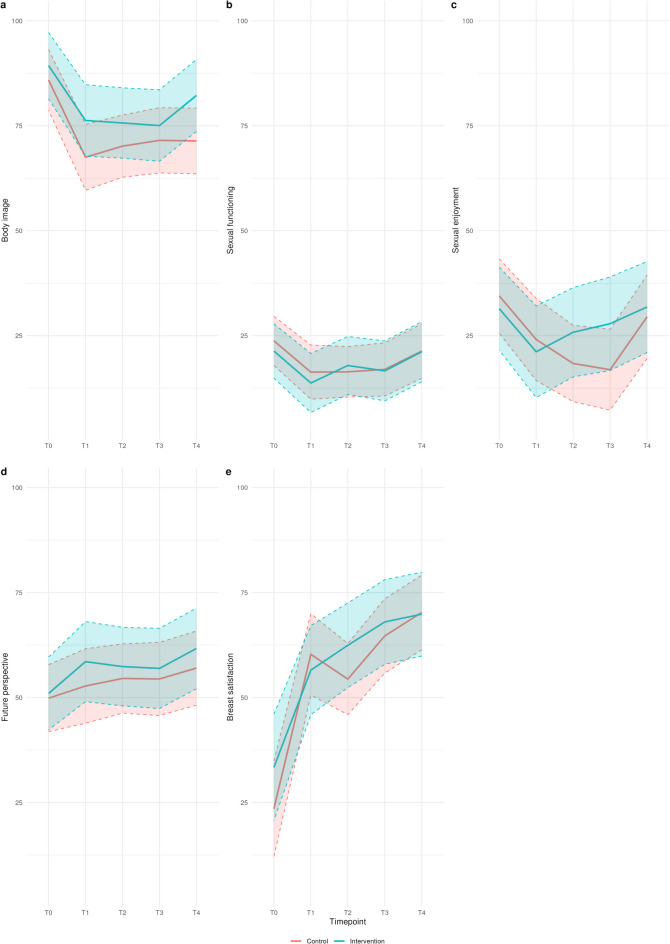
Effect plots of functional scales and items of the EORTC QLQ-BR45 for a participant with the most common baseline characteristics (primary tumor stage). T0; baseline, T1; 6 weeks, T2; 3 months, T3; 6 months, T4; 1 year from diagnosis

The body image scale at T4 was significantly higher for participants in the intervention group compared to those in the control group (*p* = 0.036, Table 1 and Appendix 1, Fig. 1). There were no statistically significant differences in the other functional scales and items of the EORTC QLQ-BR45 between the intervention and control groups (*p* > 0.05, Table 1 and Appendix 1, Fig. 1).

**Table 1:** Effect of the digital tool on functional scales and items of the EORTC QLQ-BR45

Variable	Body image		Sexual functioning		Sexual enjoyment		Future perspective		Breast satisfaction	
	*Estimates*	*p*	*Estimates*	*P*	*Estimates*	*P*	*Estimates*	*p*	*Estimates*	*p*
Intercept	85.92	** < 0.001**	23.78	** < 0.001**	34.49	** < 0.001**	49.86	** < 0.001**	23.53	** < 0.001**
T1	− 18.42	** < 0.001**	− 7.46	**0.006**	− 10.39	**0.014**	2.90	0.447	36.77	** < 0.001**
T2	− 15.77	** < 0.001**	− 7.38	**0.002**	− 16.11	** < 0.001**	4.70	0.164	30.87	** < 0.001**
T3	− 14.39	** < 0.001**	− 6.81	**0.009**	− 17.59	** < 0.001**	4.57	0.216	41.16	** < 0.001**
T4	− 14.53	** < 0.001**	− 2.43	0.368	− 4.97	0.248	7.17	0.058	46.76	** < 0.001**
T0: intervention	3.43	0.462	− 2.43	0.523	− 3.04	0.601	1.11	0.830	9.83	0.224
T1: intervention	8.76	0.092	− 2.59	0.548	− 2.93	0.658	5.80	0.328	− 3.76	0.575
T2: intervention	5.51	0.262	1.51	0.709	7.43	0.230	2.83	0.610	8.05	0.176
T3: intervention	3.52	0.492	− 0.36	0.933	10.96	0.100	2.52	0.666	3.33	0.584
T4: intervention	10.84	**0.036**	− 0.17	0.968	2.32	0.727	4.67	0.429	− 0.42	0.945
Observations	554		544		470		556		399	

T0; baseline, T1; 6 weeks, T2; 3 months, T3; 6 months, T4; 1 year from diagnosis

Fixed effects are the effect of the primary tumor stage, timepoint, and digital tool usage, and the grouping factor is participant ID with a random intercept. The reference category for the study group is the control group

Higher scores represent higher QoL

Bold indicates *P* < 0.05

When incorporating timepoint as a random effect, the intervention effect on body image at T4 remained significant (Appendix 2, Table 4).

#### EORTC QLQ-BR45: symptom scales and items

**Fig. 2:**
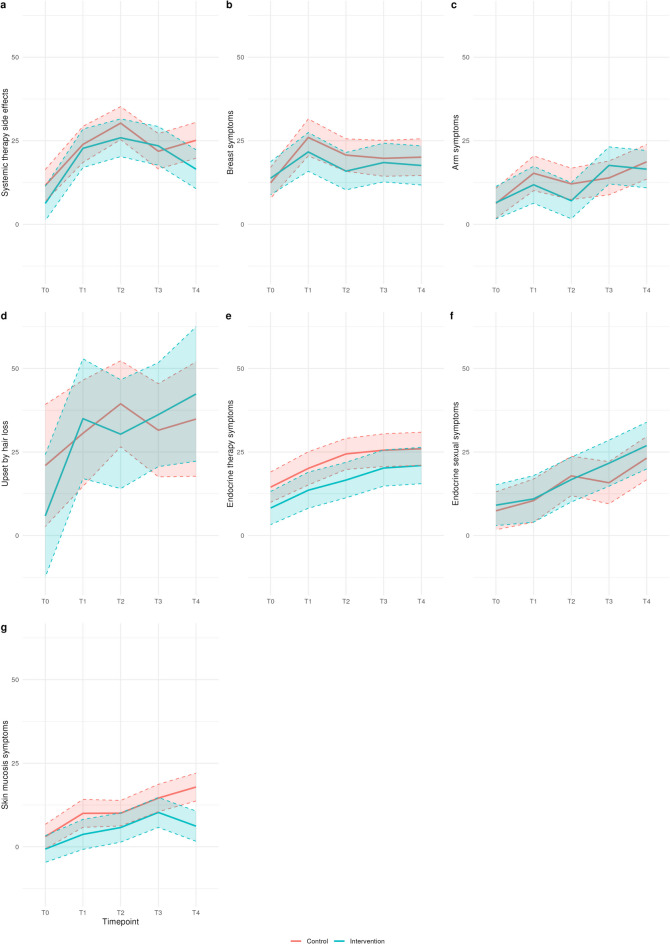
Effect plots of symptom scales and items of the EORTC QLQ-BR45 for a participant with the most common baseline characteristics (primary tumor stage). T0; baseline, T1; 6 weeks, T2; 3 months, T3; 6 months, T4; 1 year from diagnosis. Higher scores indicate more severe symptoms

Systemic therapy side effects at T4 were significantly lower for participants in the intervention group compared to those in the control group (*p* = 0.017, Table 2 and Appendix 1, Fig. 2). Endocrine therapy symptoms at T0, T1, and T2 were significantly lower for participants in the intervention group compared to the control group (*p* = 0.033, *p *= 0.049, *p* = 0.012, Table 2 and Appendix 1, Fig. 2). Skin mucosis symptoms were significantly lower at T1 and T4 for participants in the intervention group compared to those in the control group (*p* = 0.027, *p* < 0.001, Table 2 and Appendix 1, Fig. 2).

**Table 2:** Effect of the digital tool on symptom scales and items of the EORTC QLQ-BR45

Variable	Systemic therapy side effects		Breast symptoms		Arm symptoms		Upset by hair loss		Endocrine therapy symptoms		Endocrine sexual symptoms		Skin mucosis symptoms	
	*Estimates*	*p*	*Estimates*	*p*	*Estimates*	*p*	*Estimates*	*p*	*Estimates*	*P*	*Estimates*	*p*	*Estimates*	*p*
Intercept	11.62	** < 0.001**	12.41	** < 0.001**	6.26	**0.007**	20.95	**0.027**	14.48	** < 0.001**	7.41	**0.011**	3.05	0.103
T1	12.29	** < 0.001**	13.59	** < 0.001**	9.02	**0.001**	9.64	0.357	5.61	**0.006**	3.05	0.340	6.95	**0.001**
T2	18.65	** < 0.001**	8.31	**0.002**	5.84	**0.011**	18.47	**0.047**	9.94	** < 0.001**	10.45	** < 0.001**	6.99	** < 0.001**
T3	10.23	** < 0.001**	7.33	**0.014**	7.62	**0.002**	10.55	0.270	11.06	** < 0.001**	8.39	**0.007**	11.53	** < 0.001**
T4	13.51	** < 0.001**	7.69	**0.011**	12.45	** < 0.001**	13.90	0.201	11.48	** < 0.001**	15.76	** < 0.001**	14.82	** < 0.001**
T0: intervention	− 5.37	0.083	1.38	0.657	0.21	0.944	− 15.07	0.225	− 6.25	**0.033**	1.68	0.653	−3.72	0.122
T1: intervention	− 1.17	0.749	− 4.34	0.258	− 3.44	0.331	4.37	0.695	− 6.53	**0.049**	0.51	0.908	−6.29	**0.027**
T2: intervention	− 4.38	0.194	− 4.81	0.162	− 5.05	0.119	− 9.10	0.308	− 7.83	**0.012**	−1.11	0.784	−4.28	0.101
T3: intervention	1.63	0.649	− 1.25	0.737	3.75	0.279	4.66	0.612	− 5.34	0.102	5.85	0.174	−4.30	0.122
T4: intervention	− 8.65	**0.017**	− 2.49	0.512	− 2.20	0.531	7.50	0.547	− 5.02	0.129	3.75	0.395	−11.71	** < 0.001**
Observations	561		558		558		212		558		549		559	

T0; baseline, T1; 6 weeks, T2; 3 months, T3; 6 months, T4; 1 year from diagnosis

Fixed effects are the effect of the primary tumor stage, timepoint, and digital tool usage, and the grouping factor is participant ID with a random intercept. The reference category for the study group is the control group

Higher scores indicate more severe symptoms

Bold indicates *P* < 0.0

When adding timepoint as a random effect, the intervention effects for systemic therapy side effects, endocrine therapy symptoms, and skin mucosis symptoms remained statistically significant. Additionally, systemic therapy side effects and skin mucosis symptoms at T0 were significantly lower for participants in the intervention group compared to the control group (Appendix 2, Table 5).

#### HADS scores

**Fig. 3:**
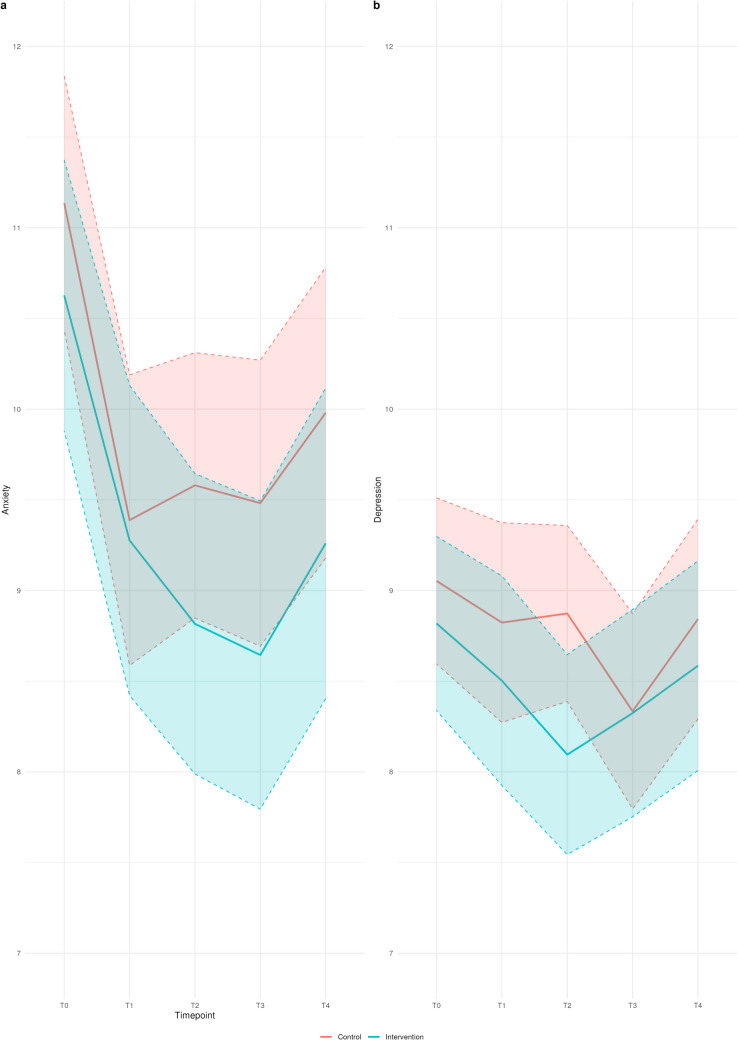
Effect plots of HADS scores for a participant with the most common baseline characteristics (primary tumor stage). T0; baseline, T1; 6 weeks, T2; 3 months, T3; 6 months, T4; 1 year from diagnosis. Higher total scores are indicative of more psychological distress

The depression score at T2 was significantly lower for participants in the intervention group compared to those in the control group (*p* = 0.024, Table 3 and Appendix 1, Fig. 3). There were no statistically significant differences in anxiety scores between the intervention and control groups (*p* > 0.05, Table 3 and Appendix 1, Fig. 3).

**Table 3:** Effect of the digital tool on HADS scores

Variable	Anxiety		Depression	
	*Estimates*	*p*	*Estimates*	*p*
Intercept	11.14	** < 0.001**	9.05	** < 0.001**
T1	− 1.75	** < 0.001**	−0.23	0.458
T2	− 1.56	** < 0.001**	−0.18	0.515
T3	− 1.65	** < 0.001**	−0.72	**0.017**
T4	− 1.16	**0.002**	−0.21	0.494
T0: intervention	− 0.51	0.263	−0.23	0.442
T1: intervention	− 0.11	0.838	−0.32	0.404
T2: intervention	− 0.76	0.127	−0.78	**0.024**
T3: intervention	− 0.84	0.116	−0.01	0.978
T4: intervention	− 0.72	0.183	−0.26	0.498
Observations	568		568	

T0; baseline, T1; 6 weeks, T2; 3 months, T3; 6 months, T4; 1 year from diagnosis

Fixed effects are the effect of the primary tumor stage, timepoint, and digital tool usage, and the grouping factor is participant ID with a random intercept. The reference category for the study group is the control group

Higher total scores are indicative of more psychological distress [12, 18, 19]

Bold indicates *P* < 0.05

When adding timepoint as a random effect, the intervention effect for the depression score at T2 remained statistically significant (Appendix 2, Table 6).

## Conclusion

Linear mixed effect modelling demonstrated generally no significant differences in the functional scales and items of the EORTC QLQ-BR45 between participants in the intervention and control groups when adjusted for the primary tumor stage. However, participants in the intervention group scored significantly lower on certain symptom scales and items of the EORTC QLQ-BR45. It is important to note that the participants in the intervention group already had significantly lower scores on the symptom scales and items at baseline (T0), which indicates that these participants might have been less symptomatic even before using the digital tool. Additionally, the HADS scores assessing psychological distress showed no significant differences between the two groups.

Consequently, it may be concluded that the breast cancer digital tool did not have a statistically significant effect on breast cancer-specific HRQoL and psychological distress over time compared to standard care alone in participants with early-stage breast cancer. The results of the current report are consistent with the findings of the primary ADAPT paper, which demonstrated that the digital tool did not have a significant effect on patient activation, HRQoL, and health status [9].

However, it is important to highlight the high level of non-adherence observed in the intervention group, as mentioned in the primary article [9]. This underscores the reality that participants do not automatically engage with a digital health app, suggesting that there are potential barriers to digital tool usage [20]. In a ‘real-world’ setting, spontaneous digital tool usage may even be less common compared to the trial setting. Thus, before the widespread implementation of digital tools in breast cancer healthcare, in-depth research is necessary to identify and mitigate the barriers to effective digital tool usage [21, 22].

One possible explanation for the high level of non-adherence could be the timing of the tool’s introduction (at diagnosis and before starting treatment), when patients are likely to feel overwhelmed by medical information and emotional distress [23]. Additionally, studies with post-treatment follow-up have shown promising effects, indicating that digital tools may be better suited for long-term care management or for patients who have completed active treatment [24].

Another explanation could be the study design, as it could be questioned whether RCTs are the optimal study design for examining digital tools. Participants in the ADAPT study were not regularly reminded to use the digital tool to avoid bias. Similarly, the results of another RCT aiming to examine the efficacy of an online decision aid for breast cancer patients considering immediate reconstruction showed no significant differences in the primary outcome of decisional conflict between the intervention and control groups [25]. An observational study design, where patients are actively reminded and encouraged to use digital tools, might have been more suitable.

Lastly, the digital offered personalized, but not necessarily tailored, medical information. To improve future digital health interventions, these tools should better meet patients’ individual care needs and preferences, considering, for example, their timeline, stage, and psychological status [26].

These findings do not imply that app-based interventions are inherently ineffective; however, they demonstrate that their success depends heavily on multiple factors, such as the context in which they are introduced, how well they align with patients’ needs, and the level of engagement they facilitate.

From a healthcare system perspective, digital tools may serve as a complementary resource, especially when personalized and well-integrated into care [27–29]. Further research is needed to define which patient populations benefit most and how digital solutions can be implemented efficiently [21, 22].

Currently, a quantitative study with the participants from the ADAPT study is being conducted to gain insights into user experiences, including potential barriers such as digital health literacy and technical issues. The results of that study can help refine digital tools to better support individual breast cancer patients and to identify which patients might benefit the most.

## Data Availability

No datasets were generated or analysed during the current study.

## Appendix 2

Tables 4, 5 and 6

**Table 4:** Effect of the digital tool on functional scales and items of the EORTC QLQ-BR45

Variable	Body image		Sexual functioning		Sexual enjoyment		Future perspective		Breast satisfaction	
	*Estimates*	*p*	*Estimates*	*p*	*Estimates*	*p*	*Estimates*	*p*	*Estimates*	*p*
Intercept	85.64	** < 0.001**	23.59	** < 0.001**	35.02	** < 0.001**	49.87	** < 0.001**	20.94	**0.002**
T1	− 19.27	** < 0.001**	− 7.12	**0.002**	− 10.97	**0.009**	1.63	0.685	41.50	** < 0.001**
T2	− 15.46	** < 0.001**	− 7.33	**0.001**	− 15.68	** < 0.001**	4.67	0.179	35.38	** < 0.001**
T3	− 15.29	** < 0.001**	− 6.46	**0.020**	− 17.98	** < 0.001**	4.11	0.317	45.41	** < 0.001**
T4	− 13.90	** < 0.001**	− 1.79	0.560	− 5.93	0.181	7.01	0.063	50.68	** < 0.001**
T0: intervention	3.62	0.342	− 2.34	0.556	− 2.54	0.679	0.84	0.874	11.87	0.211
T1: intervention	9.43	0.097	− 2.86	0.434	− 0.69	0.914	6.24	0.283	− 3.81	0.582
T2: intervention	3.79	0.485	1.34	0.731	7.05	0.212	2.41	0.652	7.17	0.252
T3: intervention	4.62	0.391	− 0.40	0.924	10.02	0.102	3.37	0.574	3.85	0.454
T4: intervention	10.46	**0.043**	− 0.62	0.898	4.59	0.520	4.71	0.404	− 0.89	0.875
Observations	554		544		470		556		399	

T0; baseline, T1; six weeks, T2; three months, T3; six months, T4; one year from diagnosis

Fixed effects are the effect of the primary tumor stage and digital tool usage; random effect is the effect of timepoint, and the grouping factor is participant ID. The reference category for the study group is the control group

Higher scores represent higher QoL

Bold indicates *P* < 0.05

**Table 5:** Effect of the digital tool on symptom scales and items of the EORTC QLQ-BR45

Variable	Systemic therapy side effects		Breast symptoms		Arm symptoms		Upset by hair loss		Endocrine Therapy symptoms		Endocrine sexual symptoms		Skin mucosis symptoms	
	*Estimates*	*p*	*Estimates*	*p*	*Estimates*	*p*	*Estimates*	*p*	*Estimates*	*p*	*Estimates*	*p*	*Estimates*	*p*
Intercept	13.16	** < 0.001**	11.89	** < 0.001**	6.41	**0.001**	11.44	0.102	14.82	** < 0.001**	8.17	** < 0.001**	4.04	**0.001**
T1	13.04	** < 0.001**	13.45	** < 0.001**	8.97	**0.001**	19.43	0.086	6.02	**0.001**	2.49	0.279	7.58	** < 0.001**
T2	18.42	** < 0.001**	8.27	**0.007**	5.75	**0.008**	29.10	** < 0.001**	9.93	** < 0.001**	10.09	** < 0.001**	6.96	** < 0.001**
T3	10.23	** < 0.001**	7.23	**0.011**	8.18	** < 0.001**	22.76	**0.004**	11.72	** < 0.001**	8.90	**0.006**	12.39	** < 0.001**
T4	13.67	** < 0.001**	8.11	**0.002**	11.69	** < 0.001**	18.73	** < 0.001**	11.50	** < 0.001**	15.25	** < 0.001**	14.76	** < 0.001**
T0: intervention	− 5.51	**0.023**	0.89	0.746	− 0.38	0.879	− 5.47	0.579	-6.72	**0.009**	1.03	0.719	-3.91	**0.009**
T1: intervention	− 1.25	0.756	− 5.19	0.225	− 3.86	0.328	4.97	0.608	-7.25	**0.028**	0.54	0.886	-6.24	**0.046**
T2: intervention	− 3.31	0.408	− 5.38	0.148	− 5.43	0.068	− 12.19	0.142	-8.17	**0.010**	-1.02	0.803	-4.20	0.104
T3: intervention	1.40	0.710	− 1.79	0.620	3.83	0.310	1.43	0.875	-6.12	0.100	4.96	0.273	-5.28	0.152
T4: intervention	− 9.02	**0.009**	− 2.83	0.427	− 2.17	0.598	17.34	0.098	-5.20	0.154	4.25	0.475	-10.60	**0.002**
Observations	561		558		558		212		558		549		559	

T0; baseline, T1; 6 weeks, T2; 3 months, T3; 6 months, T4; 1 year from diagnosis

Fixed effects are the effect of the primary tumor stage and digital tool usage; random effect is the effect of timepoint, and the grouping factor is participant ID. The reference category for the study group is the control group

Higher scores indicate more severe symptoms

Bold indicates *P *< 0.05

**Table 6:** Effect of the digital tool on HADS scores

Variable	Anxiety		Depression	
	*Estimates*	*p*	*Estimates*	*p*
Intercept	11.11	** < 0.001**	9.03	** < 0.001**
T1	− 1.67	** < 0.001**	-0.24	0.514
T2	− 1.58	** < 0.001**	-0.20	0.466
T3	− 1.64	** < 0.001**	-0.72	**0.028**
T4	− 1.13	**0.004**	-0.16	0.595
T0: intervention	− 0.51	0.281	-0.25	0.458
T1: intervention	− 0.26	0.638	-0.45	0.267
T2: intervention	− 0.66	0.171	-0.80	**0.010**
T3: intervention	− 0.70	0.212	-0.03	0.939
T4: intervention	− 0.71	0.159	-0.34	0.268
Observations	568		568	

T0; baseline, T1; six weeks, T2; three months, T3; six months, T4; one year from diagnosis

Fixed effects are the effect of the primary tumor stage and digital tool usage; random effect is the effect of timepoint, and the grouping factor is participant ID. The reference category for the study group is the control group

Higher total scores are indicative of more psychological distress

Bold indicates *P* < 0.05
